# Emotional Brain Development: Neurobiological Indicators from Fetus Through Toddlerhood

**DOI:** 10.3390/brainsci15080846

**Published:** 2025-08-08

**Authors:** Gerry Leisman, Rahela Alfasi, Amedeo D’Angiulli

**Affiliations:** 1 Movement and Cognition Laboratory, Department of Physical Therapy, University of Haifa, Haifa 3498838, Israel; rahela.arigo@gmail.com; 2 Resonance Therapeutics Laboratory, Department of Neurology, University of the Medical Sciences of Havana, Havana 11600, Cuba; 3 Center for the Developing Child, Rockville Centre, NY 11570, USA; 4 Department of Neuroscience, Carleton University, Ottawa, ON K1S 5B6, Canada; amedeo.dangiulli@carleton.ca; 5 Children’s Hospital of Eastern Ontario Research Institute, Ottawa, ON K1H 8L1, Canada

**Keywords:** human development, emotional development, cognition, brain maturation, neuroplasticity, maternal stress

## Abstract

The maturation of brain regions involved in emotion regulation—particularly the amygdala and prefrontal cortex—from fetal life to age two is a dynamic process shaped by genetic and environmental factors. Early experiences, especially responsive caregiving, promote the growth of neural circuits supporting emotional expression and regulation. In contrast, early adversity such as neglect or chronic stress may disrupt these circuits and increase vulnerability to emotional difficulties. Elevated levels of placental CRH are linked to alterations in fetal brain development related to emotion. Neurodevelopmental processes like synaptic pruning and myelination, active during the first years, further shape emotional circuitry. These findings underscore the importance of early caregiving and timely interventions in fostering healthy emotional development. The present article proposes an integrative conceptual framework for early emotional and cognitive development, combining neurobiological models with contemporary theories in developmental psychology.

## 1. Introduction

The maturation of brain regions associated with emotion from fetal development through the age of two is a complex and dynamic process. This period is marked by significant growth and differentiation in neural circuits that are crucial for emotional regulation and expression. Early experiences and environmental factors play a critical role in shaping these developing brain regions, influencing the trajectory of emotional development. Newborns initially exhibit a limited range of discrete emotional expressions. However, as they grow, they display a broader spectrum of emotions and become more responsive to a variety of eliciting conditions [1]. This expansion in emotional expression is closely linked to the maturation of specific brain regions, such as the amygdala and prefrontal cortex, involved in the processing and regulation of emotions, along with cumulative exposure to an environment that requires the processing and learning and memorizing of emotional content [2].

This article presents a unified perspective on early emotional and cognitive development, integrating insights from neuroscience and developmental psychology. It describes the infant brain as a dynamic system shaped by early interactions with caregivers and rooted in innate emotional systems [3,4], which provide the basis for attachment and self-regulation [3]. Feldman’s biopsychosocial model [5,6] suggests that the development of the social brain occurs through time-sensitive “windows of opportunity,” shaped by reciprocal interactions such as emotional synchrony, touch, and vocal exchange. Damasio’s [7] Affective Core Theory emphasizes the central role of embodied emotional experience in shaping the sense of self and the foundations of cognition. In addition, the predictive processing framework offers a unifying lens through which the brain is viewed as an active predictive agent, continually updating its internal models based on gaps between expectations and sensory input. Early learning, then, involves minimizing prediction errors, with key structures such as the hippocampus and prefrontal cortex supporting the integration of memory, attention, and sensory processing. Together, these perspectives highlight how neural development, emotional experience, and predictive learning interact as part of a dynamic process that shapes both the developing brain and the emerging self.

Learning and memory begin before birth. By around 18 weeks of gestation, critical brain structures like the cortex and hippocampus start forming. The hippocampus, essential for memory formation and consolidation, continues developing after birth and integrates with the frontal lobes to support cognitive functions. The prefrontal cortex, part of the frontal lobes, develops in utero and matures well into childhood, supporting executive functions such as attention regulation, planning, and decision-making. It enables infants to focus, filter stimuli, and anticipate outcomes. Sleep plays a crucial role during infancy, supporting neural reorganization and memory consolidation. Additionally, the association cortices in the temporal and parietal lobes store detailed experiential information, enriching early learning [4].

The maturation of these brain regions is not exclusively dictated by genetic factors but is also profoundly affected by the infant’s surroundings. Nurturing and responsive caregiving have been demonstrated to enhance the development of brain circuits linked to emotional control. This form of caring delivers essential stimulation and support for the appropriate development of these brain regions, hence fostering healthy emotional maturation [8,9].

In contrast, negative early events, including neglect or persistent stress exposure, can adversely affect the growing brain. These negative experiences can disrupt the maturation of neural circuits, leading to difficulties in emotional regulation and increased vulnerability to mental health issues later in life [10,11]. The interplay between genetic predispositions and environmental influences underscores the importance of early interventions and supportive caregiving practices to foster healthy emotional development.

Research has also highlighted the role of placental CRH in fetal neurodevelopment. Elevated levels of placental CRH have been associated with alterations in brain development that can affect emotional regulation [9]. This finding emphasizes the need to consider prenatal factors when examining the early foundations of emotional health. Furthermore, the first years of life are characterized by rapid changes in the brain’s structure and function. During this period, the brain undergoes significant synaptic pruning and myelination, processes that are essential for the efficient functioning of neural circuits involved in emotion [12].

Additionally, the transfer of early childhood experiences to biological markers during critical periods of emotional development is significantly influenced by various epigenetic mechanisms. These mechanisms, particularly DNA methylation and histone modifications, play a crucial role in how early-life stressors shape long-term behavioral and emotional outcomes. Among the key epigenetic mechanisms that can shape long-term emotional development include DNA methylation, a stable form of epigenetic change that alters gene expression without modifying the DNA sequence. It has been linked to the regulation of stress-response genes, such as NR3C1 and FKBP5, which are crucial for the hypothalamus–pituitary–adrenal (HPA) axis function [13]. Histone modifications can also affect the chromatin structure, influencing gene accessibility and expression. They are responsive to environmental factors, including maternal care and nutrition, which are vital during sensitive developmental periods [14]. Also, biological embedding can occur through adversities in the first few years of life that can lead to lasting physiological changes, establishing a foundation for stress vulnerability and psychopathology later in life [15].

While the focus is often on negative experiences, positive early interactions can also induce beneficial epigenetic changes, highlighting the dual potential of early childhood experiences in shaping biological and psychological outcomes.

These neurodevelopmental changes are influenced by both intrinsic genetic programs and extrinsic environmental factors, highlighting the complex interplay between nature and nurture in shaping emotional development. In summary, the maturation of brain regions associated with emotion from fetal development to the age of two is a multifaceted process influenced by a combination of genetic, environmental, and caregiving factors. Understanding these influences is crucial for developing strategies to support healthy emotional development and mitigate the impact of adverse early experiences. The insights gained from this research can inform interventions aimed at promoting emotional well-being from the earliest stages of life [1,2,5,9].

## 2. Background and Significance

### 2.1. Historical Perspectives on Emotional Brain Development

The historical perspectives on emotional brain development have evolved significantly over the years, with early research primarily focusing on the anatomical and functional maturation of brain regions associated with emotion. Initial studies highlighted the importance of the prenatal environment in shaping the brain’s structural and functional connectivity. For instance, prenatal maternal stress has been shown to have lasting effects on brain development, including alterations in regional brain volumetric growth, cortical folding, and functional connectivity [16]. These early findings laid the groundwork for understanding how prenatal factors influence emotional development.

Further research emphasized the critical period of brain development during infancy and early childhood. This period is marked by rapid maturation of neural substrates that underpin cognitive and behavioral functions. Studies have demonstrated that the early environment, including factors such as stress and cognitive enrichment, can significantly impact the pace and trajectory of brain development. High stress and low cognitive enrichment have been associated with accelerated changes in cortical thickness and surface area, as well as shifts in functional network segregation [17]. These changes can affect the brain’s plasticity, influencing emotional and cognitive outcomes.

The development of socioemotional skills and social cognition also occurs predominantly before the age of six, underscoring the importance of early experiences in shaping the social brain. Connecting alterations in brain substrates to behavioral development is essential for comprehending the mechanisms that govern social interactions and relationships [18]. This period of development is particularly sensitive, and early interventions can have profound effects on long-term emotional health.

Research on the functional connectivity of the brain has revealed that the anatomical structure of the cerebral cortex constrains functional connectivity. Investigating the relationships between neonatal white matter diffusion characteristics and later socioemotional outcomes has provided insights into how the brain’s emerging architecture influences development, particularly in very preterm children [10]. These findings highlight the importance of early brain structure in shaping emotional and cognitive trajectories.

The role of caregiving in emotional development has also been a focal point of research. Infants carry internal representations of past interactive experiences, which influence their affective and interactive styles. This process can be conceptualized in terms of developing cortical networks during the first two years of life [19]. Responsive and nurturing caregiving is essential for healthy emotional development, as it supports the establishment of these critical neural networks.

Temperamental variations among infants further illustrate the complexity of emotional development. Differences in emotional makeup, such as reactivity and irritability, are often described in terms of temperament. For example, highly reactive and irritable babies are frequently labeled as “difficult,” while those prone to positive emotions and less reactivity are considered “easygoing” [1]. These temperamental differences can influence how children respond to their environments and caregivers, affecting their emotional development.

Historical perspectives on emotional brain development have evolved from a focus on anatomical and functional maturation to a deeper understanding of how environmental factors and relationships shape early neurodevelopment. Early research emphasized the influence of the prenatal environment, showing that maternal stress can lead to lasting changes in brain structure and function such as changes in regional volume, cortical folding, and connectivity [20]. Importantly, stress is not a uniform phenomenon; it varies in intensity, duration, and relative context. A distinction is usually made between acute (short-term), chronic (long-term), and toxic stress, defined as the prolonged activation of stress responses in the absence of protective relationships.

In care settings, stress may stem from socioeconomic hardship, mental health conditions, or social isolation, and is typically assessed using biomarkers (e.g., cortisol), behavioral observations, or self-reports. While tolerable stress can promote adaptive coping and resilience, chronic or toxic stress may impair the development of central control systems, particularly within the amygdala, hippocampus, and prefrontal cortex. These insights highlight the dynamic interplay between prenatal and postnatal experiences and the formation of emotional circuits in the brain.

### 2.2. Importance of Early Brain Development

The importance of early brain development cannot be emphasized, as it establishes the framework for a child’s cognitive and emotional health. During the first two years of life, the brain undergoes significant structural and functional changes, particularly in regions associated with emotion regulation and social processing. These changes are influenced by both genetic and environmental factors, including the quality of caregiving and exposure to stress.

Research has shown that the brain is highly malleable during early development, making it particularly sensitive to environmental influences. This malleability involves multiple neurobiological mechanisms, including synaptic pruning, in which redundant neural connections are removed to improve efficiency; myelination, which improves the speed and fidelity of neural communication; and experience-dependent reorganization, whereby neural circuits adapt based on sensory and social experiences. For example, the hippocampus, a brain region involved in regulating the hypothalamic–pituitary–adrenal (HPA) axis, is significantly affected by early-life stress and adversity, which can alter synaptic density and patterns of myelination. Positive experiences, such as nurturing and responsive care, promote healthy synaptic growth and refinement of circuits critical for emotional development.

In addition, fronto-limbic and temporal circuits, which are essential for emotional processing and emotion regulation, show significant structural and functional alterations following exposure to postnatal stress [21]. Animal studies demonstrate how enriched environments enhance dendritic complexity and synaptic strength in these regions, whereas deprivation or stress can lead to reduced connectivity and impaired emotional regulation. These circuits are key to the evaluation of social stimuli and the management of emotional responses, highlighting the central role of early experiences in shaping the architecture and function of the brain.

Experience-dependent reorganization is another key mechanism highlighted in animal models. For example, maternal care in rats influences the development of fronto-limbic circuits by modulating glucocorticoid receptor expression, which, in turn, affects stress reactivity and emotional regulation [22]. Similarly, early-life stress paradigms in rodents show alterations in synaptic connectivity and myelination patterns in the amygdala and hippocampus, correlating with behavioral changes in anxiety and social interaction [23].

Caregiving-focused interventions during infancy have demonstrated long-term positive effects on brain structure and activity, as well as on the regulation of the HPA axis. For example, high-quality maternal care has been shown to decrease cortisol reactivity and promote faster recovery from stress, indicating the profound impact of nurturing caregiving on the developing brain [24].

Furthermore, the development of white matter tracts in preterm infants has been linked to socioemotional development. Studies utilizing advanced imaging techniques, such as 3T MRI, have explored the relationship between the microstructure of these tracts and emotional outcomes in children born preterm. These findings underscore the importance of early brain development in determining future emotional health [25].

Maternal stress during pregnancy and the postpartum period can also influence brain adaptations to motherhood. Stress exposure can affect maternal brain activations related to empathy and emotion regulation, which are essential for understanding and responding to a child’s emotional needs. This highlights the need for support systems to help mothers manage stress and enhance their caregiving abilities [26].

The prenatal period is another critical window for brain development. Maternal stress during pregnancy has been associated with changes in the child’s brain before birth, affecting regions involved in emotional regulation and social processing. Psychosocial support and healthy behaviors during pregnancy can mitigate these effects, emphasizing the importance of maternal well-being for optimal fetal brain development [27].

In summary, early brain development is a complex process influenced by a myriad of factors, including genetic predispositions, environmental exposures, and the quality of caregiving. The brain’s plasticity during this period allows for significant growth and adaptation, making early experiences crucial for shaping a child’s emotional and cognitive health. Understanding these processes can inform interventions and support systems aimed at promoting healthy brain development and emotional well-being in children.

### 2.3. Overview of Emotional Brain Structures

The maturation of brain regions associated with emotion begins in the fetal stage and continues through the age of two, involving complex interactions between genetic, environmental, and experiential factors. The frontal lobe, especially the prefrontal cortex, is essential for cognitive and emotional function. Despite earlier assumptions that the frontal lobe is nonfunctional during infancy, recent research underscores its importance in early behavioral development [19].

The development of emotional brain structures is significantly influenced by maternal signals. Studies combining human and animal research have shown that patterns of maternal signals are evolutionarily conserved and crucial for the development of learning, memory, and emotional regulation systems in the brain [28]. These signals help shape the organization of the developing brain, highlighting the importance of early caregiving environments.

Stress during pregnancy can have profound effects on the developing brain. Excessive prenatal stress is associated with alterations in brain structure and function, which can have long-term implications for health across the lifespan [27]. Specifically, cortical thinning in the frontal and temporal areas has been linked to prenatal stress, which, in turn, is associated with depressive symptoms in later childhood [29]. This suggests that the prenatal environment can have lasting effects on emotional health.

Preterm birth also affects brain development differently compared to full-term birth. Exposure to the extrauterine environment and deprivation of intrauterine signals in late pregnancy can shape the infant brain in unique ways [30]. This highlights the need for in vivo studies on normally developing fetuses to better understand the link between brain anatomy and functional connectivity.

The development of socioemotional responses and cognitive functions is also influenced by maternal hormones and signaling molecules that cross the placenta. Positive maternal mood during pregnancy has been shown to have beneficial effects on the child’s brain development and function [31]. This underscores the importance of maternal well-being for optimal brain development.

Postnatal experiences continue to shape brain development. For instance, first-time mothers who reported less warmth and care from their own mothers exhibited reduced activation in brain regions involved in emotion regulation and social information processing when responding to infant cries. This suggests that early caregiving experiences can influence the neural circuits involved in emotional responses.

Furthermore, the first few months postpartum are characterized by significant changes in brain structure in mothers. Increases in gray matter volume in regions such as the striatum, amygdala, hypothalamus, and substantia nigra have been observed, which are involved in maternal motivation [26]. These changes highlight the dynamic nature of the brain in response to caregiving roles.

Longitudinal research is essential to understand how supportive postnatal environments can modify brain developmental trajectories initiated by prenatal stress exposures. Ongoing multisite studies, such as the Healthy Brain and Child Development Study, aim to address these questions and provide insights into the interplay between prenatal and postnatal factors in brain development [24].

In summary, the development of emotional brain structures is a complex process influenced by a range of factors, including prenatal stress, maternal signals, and early caregiving experiences. Understanding these influences is crucial for promoting optimal emotional health and development in children.

## 3. Neuroplasticity in Early Development

### 3.1. Mechanisms of Neuroplasticity

Mechanisms of neuroplasticity during early development are critical for understanding how the brain adapts and reorganizes in response to various stimuli and experiences. Neuroplasticity refers to the brain’s ability to change and adapt throughout life, particularly during early developmental periods when the brain is most malleable. During the third trimester of gestation and the neonatal period, significant neurodevelopmental processes occur, including the formation of cerebral pathways, pathfinding, target selection, and growth into the cortical plate [32,33]. These processes are essential for establishing the foundational architecture of the brain, which will later support more complex functions.

The influence of maternal psychological stress on fetal brain development has been well documented. Stress-sensitive aspects of maternal–placental–fetal biology play crucial roles in fetal brain development, providing mechanistic pathways for observed alterations in brain structure and function [20]. For instance, maternal distress during pregnancy can impact the fronto-amygdala circuitry, which is involved in emotional regulation and processing [34]. This circuitry’s development is particularly sensitive to environmental influences, highlighting the importance of a nurturing and responsive caregiving environment.

Neuroimaging studies have shown that early experiences, such as emotional neglect, can lead to alterations in neonatal functional connectivity between key brain regions, including the amygdala and prefrontal cortex [31,32]. These changes can have long-term implications for emotional development and psychiatric risk. For example, infants exposed to maternal childhood maltreatment exhibit stronger functional connectivity between the amygdala and regions such as the ventromedial prefrontal cortex (vmPFC) and dorsal anterior cingulate cortex (dACC) [34]. These findings underscore the lasting impact of early adverse experiences on brain development.

**Figure 1:**
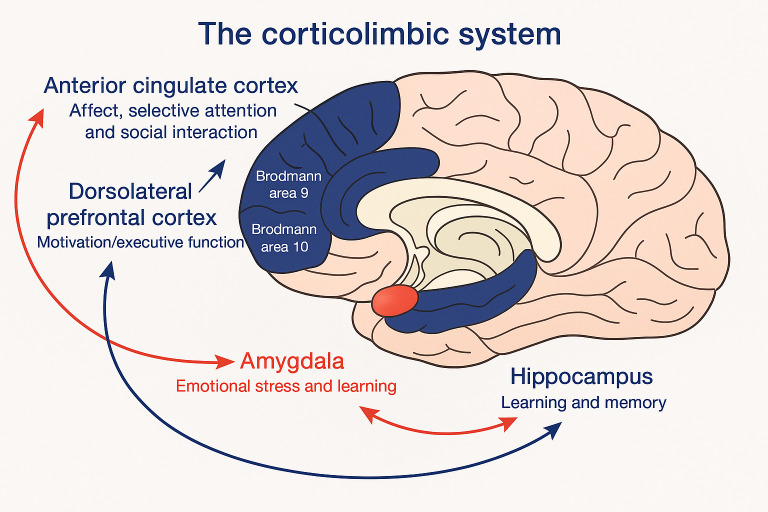
The corticolimbic system consists of several brain regions that include the rostral anterior cingulate cortex, hippocampal formation, and basolateral amygdala. The anterior cingulate cortex has a central role in processing emotional experiences at the conscious level and selective attentional responses. Emotionally related learning is mediated through the interactions of the basolateral amygdala and hippocampal formation, and motivational responses are processed through the dorsolateral prefrontal cortex (adapted from [35] with permission).

Furthermore, the first two years of life are marked by significant developmental changes in brain regions associated with emotion, particularly the limbic system. The brain undergoes widespread changes during this period, with notable developments in both limbic and subcortical areas. These changes are not linear but occur in distinct phases, with more rapid changes observed during the first year compared to the second, followed by another period of dramatic changes from ages two to six [34]. This pattern of development highlights the dynamic nature of neuroplasticity during early childhood. The role of caregiving quality in shaping brain development cannot be overstated. The limbic system connectivities are exemplified in Figure 1.

Variations in caregiving, such as differences in maltreatment and separation from caregivers, can significantly impact the biology of the developing brain [33]. These experiences can lead to structural and functional differences in critical brain circuits, such as the amygdala–hippocampal–prefrontal circuits, which are involved in fear learning and emotional regulation [34]. Understanding these mechanisms is essential for developing interventions that support healthy emotional development.

In summary, the mechanisms of neuroplasticity during early development are influenced by a complex interplay of genetic, biological, and environmental factors. The brain’s ability to adapt and reorganize in response to early experiences is crucial for emotional development and long-term mental health. Research in this area continues to uncover the intricate processes that underlie neuroplasticity, providing valuable insights into how early interventions can promote optimal brain development and emotional well-being.

### 3.2. Critical Periods of Development

Critical periods of development are essential phases during which the brain exhibits heightened plasticity, allowing for significant growth and reorganization in response to environmental stimuli. These periods are crucial for the maturation of brain regions associated with emotion, beginning from fetal development and extending through the age of two.

During the prenatal period, the fetus’ brain is highly susceptible to maternal influences. The mutual regulatory relationship between the fetus’ and mother’s physiological systems, particularly across the placenta, plays a central role in programming the stress-regulating HPA axis [36]. This dyadic system is especially active in the last trimester of pregnancy, highlighting the importance of maternal health and stress levels during this time.

Research indicates that the prenatal period is a critical time when the fetal brain is vulnerable to the effects of maternal psychological and physiological distress, including prenatal depression [21]. The influence of such prenatal exposure on neonatal neural circuit maturation, although not fully understood, underscores the need for further studies to clarify these relationships [29]. Additionally, maternal stress during pregnancy can impact brain development, which can be quantified soon after birth, before exposure to other potentially confounding influences [20].

The early postnatal period is marked by the brain’s adaptation to develop within a social context. Most parts of the social brain can be activated in infants, although there are differences in specialization, localization, and functional differentiation compared to adults [18]. The early functioning of cortical structures involved in perceiving other humans and preferential attention to conspecifics is crucial for the necessary input to developing related cortical circuitry over the first few months of life. From around 27 months of age, the use and understanding of sentences mark the beginning of the second critical period, known as ‘verbal socialization.’ During this time, the process of socialization begins anew through a different form of communication, making the developing brain particularly vulnerable to environmental stresses [37]. This period is characterized by significant growth and changes in higher-order association areas compared to primary areas, with nonlinear growth patterns observed globally and region-specific growth trajectories locally [34].

The development of the frontal brain region is intimately involved in behaviors central to self-regulation, such as means–end schemas and the ability to carry out relatively complex sequences of novel behavior, including sequences of directed gaze. This suggests that frontal lobe development is crucial for the development of emotion regulation during infancy [19]. Furthermore, experiences from a mother’s own childhood, such as emotional neglect, can influence the development of fronto-amygdala circuitry in the next generation as early as one month after birth [34].

Overall, the critical periods of development are marked by significant neuroplasticity, allowing the brain to adapt and reorganize in response to environmental stimuli. These periods are essential for the maturation of brain regions associated with emotion, highlighting the importance of nurturing and responsive caregiving in shaping a child’s emotional health.

## 4. Prenatal Development of the Emotional Brain

### 4.1. Neurogenesis and Neural Migration

Neurogenesis and neural migration are fundamental processes in the prenatal development of the emotional brain. Neurogenesis, the formation of new neurons, begins early in fetal development and continues into early childhood. This process is crucial for establishing the neural circuits that will later support emotional regulation and cognitive functions. During neurogenesis, neural progenitor cells proliferate and differentiate into various types of neurons, which then migrate to their destined locations within the brain.

Neural migration is the process by which these newly formed neurons travel from their origin in the ventricular zone to their final positions in the cortical plate. This migration is guided by a combination of genetic and environmental factors, which ensure that neurons reach their appropriate destinations and form functional connections. The precise timing and pattern of neural migration are critical for the proper development of brain regions involved in emotion, such as the amygdala and prefrontal cortex.

Nelson and colleagues [11] outline that the brain’s ability to adapt and change in response to environmental stimuli, known as neural plasticity, is particularly pronounced during early development. This plasticity allows for the fine-tuning of neural circuits through processes such as synaptic overproduction and pruning, which are essential for the maturation of the emotional brain. The interaction between genetic predispositions and environmental experiences plays a significant role in shaping these developmental processes.

Emerging evidence suggests that the early-life environment, including maternal care and stress levels, can significantly influence neurogenesis and neural migration. For instance, Ref. [32] highlights the importance of maternal psychological well-being during pregnancy, as it can impact the neurodevelopmental foundations of the fetus. This underscores the need for supportive and nurturing caregiving to promote optimal brain development.

Furthermore, Mollie Marr and colleagues [33] indicate that maternal stress during pregnancy can affect the connectivity of the infant’s amygdala, a key region involved in emotional processing. This finding suggests that prenatal stress may alter the trajectory of neural migration and the establishment of emotional circuits, potentially leading to long-term effects on emotional health.

Schneider and associates [18] state that early neural correlates of social information processing, such as face and emotion recognition, begin to emerge during infancy. These early developments are supported by the functional connectedness of brain regions established through neurogenesis and neural migration. The dynamic changes in limbic and subcortical regions during the first years of life, as observed in [34], further illustrate the critical periods of early brain development that are influenced by these processes.

In summary, neurogenesis and neural migration are essential for the prenatal development of the emotional brain. These processes are influenced by a complex interplay of genetic and environmental factors, with early experiences playing a crucial role in shaping the neural circuits that underlie emotional regulation. Understanding these mechanisms provides valuable insights into the importance of nurturing and responsive caregiving during this critical period of development.

### 4.2. Formation of Subcortical Structures

**Figure 2:**
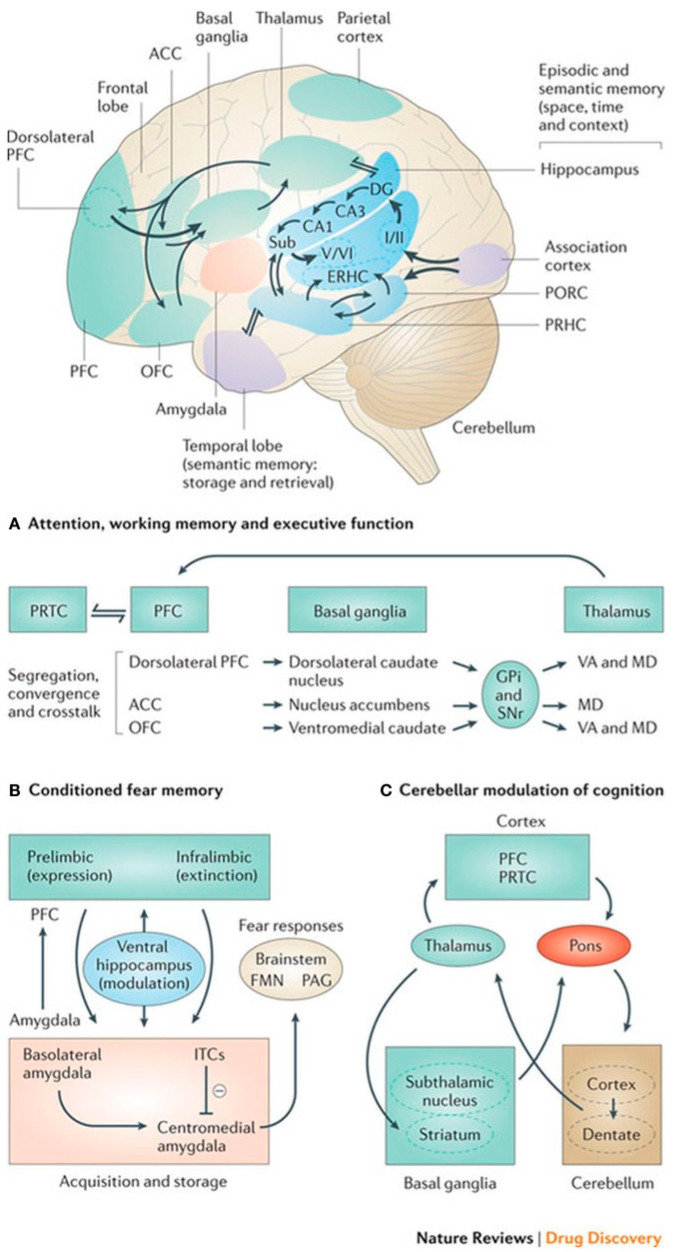
Basal ganglia connectivities to the frontal lobe for motor–cognitive interaction. The premotor and supplementary motor cortex, in particular, have specific connectivities of the basal ganglia for (**A**) attention, working memory, and executive function; (**B**) conditioned fear memory; and (**C**) cerebellar and basal ganglia modulation of cognition. All areas of the cerebral cortex project to the basal ganglia, but the output of the basal ganglia is directed toward the frontal lobe (adapted from [38] with permission).

The formation of subcortical structures during prenatal development is a critical aspect of the emotional brain’s maturation. These structures, which include the amygdala, hippocampus, and basal ganglia, play essential roles in processing emotions and regulating behavior. The development of these regions represented in Figure 2 begins early in fetal life and continues through the first few years postnatally, influenced by both genetic and environmental factors.

The amygdala, a key player in emotional processing, starts to form during the early stages of gestation. By the end of the first trimester, the basic structure of the amygdala is established, although it continues to mature and refine its connections throughout infancy and early childhood. This region is particularly sensitive to environmental influences, such as maternal stress and caregiving behaviors, which can significantly impact its development and function [12,26].

The hippocampus, another crucial subcortical structure, is involved in memory formation and emotional regulation. Its development also begins prenatally, with significant growth and differentiation occurring during the second and third trimesters. The hippocampus continues to develop postnatally, with synaptic pruning and myelination processes refining its structure and connectivity. Early experiences, including exposure to stress and the quality of maternal care, can affect hippocampal development, potentially leading to long-term implications for emotional and cognitive functions [17,38,39].

The basal ganglia, which include structures such as the caudate nucleus, putamen, and globus pallidus, are involved in motor control and various cognitive and emotional processes. These structures begin to form early in fetal development and undergo significant changes during the prenatal period. The basal ganglia’s development is influenced by genetic factors and early-life experiences, which can shape their function and connectivity. For instance, disruptions in the development of these structures have been linked to various neurodevelopmental disorders, highlighting the importance of a supportive and nurturing environment during early life [1,40].

Research using functional magnetic resonance imaging (fMRI) has provided insights into the functioning of these subcortical structures during infancy. Studies have shown that the amygdala and hippocampus are active in response to emotional stimuli, even in very young infants. These findings suggest that the basic neural circuits for emotional processing are in place early in life, although they continue to be shaped by postnatal experiences [12,33,40]. Moreover, the connectivity between subcortical structures and other brain regions, such as the prefrontal cortex, is crucial for the regulation of emotions and behavior. This connectivity develops gradually, with significant changes occurring during the first two years of life. Early experiences, including the quality of caregiving, can influence the development of these neural connections, affecting the child’s ability to regulate emotions and respond to stress [1,26,38].

In summary, the formation of subcortical structures during prenatal development is a complex process influenced by both genetic and environmental factors. The amygdala, hippocampus, and basal ganglia play essential roles in emotional processing and regulation, and their development is crucial for the overall maturation of the emotional brain. Early experiences, particularly the quality of caregiving, can have lasting effects on the structure and function of these regions, underscoring the importance of a supportive and nurturing environment during this critical period [12,34,41].

### 4.3. Cortical Development

Cortical development during the prenatal period is a complex and dynamic process that lays the foundation for emotional and cognitive functions. The development of the cortex, particularly the right hemisphere, is crucial for the child’s ability to process and respond to social and emotional stimuli. This process is significantly influenced by early experiences and environmental factors.

The right hemisphere of the brain undergoes substantial growth during the prenatal period, which is critical for the development of social–emotional capacities. The interactions between the mother and the child play a vital role in this development. The child’s right cortex uses the mother’s right cortex as a template, which helps in the hard wiring of circuits that will mediate the child’s expanding social–emotional capacities [38]. This period is marked by the imprinting of neural circuits that are essential for processing both external and internal information.

Maternal interactive style across different contexts has been shown to influence the child’s emotional, behavioral, and physiological regulation during toddlerhood. The quality of maternal interactions can affect the child’s ability to regulate emotions and behavior, which is linked to the development of the cortex [1]. This highlights the importance of nurturing and responsive caregiving in shaping a child’s emotional health.

Structural changes in the maternal brain during the postpartum period also play a role in cortical development. Studies have shown that there is a significant structural increase in many maternal brain regions involved in parenting from immediately after childbirth to 3–4 months postpartum. This structural increase is associated with a decrease in brain age among mothers, suggesting that the maternal brain undergoes changes to support the demands of parenting [26]. These changes are likely to influence the child’s cortical development through the quality of maternal care and interaction.

The ability to test causality and mechanisms in pre-clinical experimental work with animals, alongside observational longitudinal research in humans, provides compelling evidence that patterns of sensory signals during sensitive periods shape the development of neural circuits. These circuits are crucial for cognitive function and sensory processing. Early-life unpredictability has been linked to outcomes related to emotional development and mental health later in life [28]. This underscores the importance of stable and predictable environments for optimal cortical development.

The frontal lobe activity and affective behavior of infants are also influenced by maternal factors. Research has shown that changes in infant behavior are accompanied by changes in frontal EEG activity, indicating that the development of the frontal cortex is closely linked to emotional and behavioral regulation [19]. This further emphasizes the role of early experiences and maternal interactions in shaping the development of the cortex.

Child traits, like negative affectivity, may serve as early indicators of vulnerability to emotional dysregulation. Neurobiological correlates of early emotional functioning, such as neonatal amygdala resting-state connectivity, have been associated with parent-reported internalizing symptoms at age two. Additionally, DMN resting-state connectivity at birth has been negatively associated with behavioral inhibition at age two [42]. These findings suggest that early neural connectivity patterns are predictive of later emotional and behavioral outcomes, highlighting the importance of early cortical development.

A new perspective on the study of the birthing brain suggests that maternal brain changes during pregnancy prepare the brain for motherhood. This preparation likely influences the development of the child’s cortex, as the maternal brain undergoes changes that support caregiving behaviors [43]. Understanding these changes can provide insights into how maternal factors influence cortical development.

To support healthy fetal brain development, it is crucial to reduce maternal psychological stress and associated inflammation. Integrating intervention and prevention research with initiatives that advance understanding of mechanistic pathways through which early-life conditions influence neurodevelopment is essential. This approach is important from both a scientific and health systems perspective, as it can help identify effective psychotherapeutic interventions [20].

The relationship between thalamocortical and cortico-cortical connectivity across the cortex during the second and third trimesters has been investigated. Functional thalamocortical connectivity increases during gestation, and its development pattern is similar in homologous regions of both hemispheres [30]. This connectivity is essential for the development of cortical circuits that underlie emotional and cognitive functions.

Although the most dramatic structural brain development occurs during the first two years of life, the period from 2 to 4 years of age also features significant behavioral changes. This suggests that cortical development continues to be dynamic and responsive to environmental influences during early childhood [34]. Understanding these developmental trajectories can provide insights into how early experiences shape the emotional brain.

Finally, characterizing the typical development of neural circuits during infancy is crucial for understanding alterations associated with psychiatric risk. Future work with task fMRI can advance our understanding of the functioning of neural circuits during infancy and their impact on emotional and cognitive development [12]. This research can inform interventions aimed at promoting healthy cortical development and emotional health.

In summary, cortical development during the prenatal period is influenced by a complex interplay of genetic, environmental, and maternal factors. Early experiences and caregiving quality play a crucial role in shaping the neural circuits that underlie emotional and cognitive functions. Understanding these processes is essential for promoting optimal emotional health and development.

### 4.4. Influence of Maternal Factors

Maternal stress and anxiety during pregnancy have profound implications for the prenatal development of the emotional brain. Research indicates that maternal stress can influence the structural and functional integrity of brain circuits involved in emotional regulation, affective processing, and sensory processing. For instance, prenatal distress has been associated with changes in the uncinate fasciculus, cingulum, fornix, and inferior fronto-occipital fasciculus, which are critical for emotional and sensory processing [21].

The impact of maternal stress is not limited to structural changes but also extends to functional outcomes. Studies have shown that maternal perinatal stress clusters are significantly associated with the development of negative affect in infants. This relationship is evident from as early as three months and continues to influence emotional development up to 24 months of age [33]. The presence of maternal stress during pregnancy can lead to heightened brain responses to infant distress and reduced responses to positive cues, potentially impairing a mother’s ability to manage her emotions and respond sensitively to her infant’s needs.

Experimental research supports the causal link between maternal stress and changes in the maternal brain. Cross-species studies, particularly those involving rodents, have demonstrated that poor quality maternal care received in childhood can have lasting effects on stress responses and maternal outcomes in humans. This evidence underscores the importance of addressing maternal stress to improve both maternal and infant health outcomes.

Furthermore, the intrauterine environment plays a crucial role in fetal brain development. Stress-related biological signals can induce structural and functional changes in fetal cells, tissues, and organ systems during critical periods of rapid cellular proliferation and differentiation. These changes can have long-term or permanent consequences, particularly for the fetal brain, which is highly susceptible to environmental perturbations during neurodevelopmental processes such as neuron proliferation, migration, and synaptogenesis [43].

The influence of maternal stress on infant emotional development is also evident in the early postnatal period. Factors such as genetics and prenatal environment contribute to the variance in neural circuitry and negative affectivity observed shortly after birth. This highlights the critical nature of early interventions to mitigate the adverse effects of maternal stress on infant emotional development [44].

Intervention studies have shown promising results in reducing maternal psychological stress and improving maternal brain responses to infants. Programs focusing on parenting skills, self-care, and emotion regulation have demonstrated positive impacts on both maternal and infant outcomes. These findings suggest that targeted interventions during the perinatal period can enhance maternal caregiving behaviors and support healthy emotional development in infants [26].

In summary, maternal stress and anxiety during pregnancy have significant implications for the prenatal development of the emotional brain. The evidence highlights the importance of addressing maternal stress through targeted interventions to promote healthy emotional development in infants and improve maternal well-being.

### 4.5. Nutritional Influences

Nutritional influences during prenatal development play a crucial role in shaping the emotional brain of the fetus. The maternal diet provides essential nutrients that are fundamental for the proper development of fetal brain structures, which are critical for emotional regulation and reactivity.

Research indicates that maternal nutrition can significantly impact the development of the fetal limbic system, a brain region involved in emotional processing. For instance, maternal anxiety, which can be influenced by nutritional status, is associated with hyperactivation of the fetal limbic system. This hyperactivation is often accompanied by a downregulation of cortical areas responsible for higher-order cognitive functions and emotional control [32]. Such alterations in brain activity underscore the importance of adequate maternal nutrition in supporting balanced brain development.

Furthermore, exposure to low socioeconomic status (SES), which often correlates with poor nutritional intake, has been linked to changes in cortical thickness and functional segregation in young children. Cortical thickness typically peaks around the age of two years, but in children exposed to low SES, cortical thinning occurs earlier. This premature thinning is thought to reflect an earlier curtailment of synaptic proliferation and a reduced window for synaptic pruning, processes that are essential for healthy brain maturation [45]. These findings highlight the critical role of maternal nutrition in ensuring optimal brain development during early childhood.

Additionally, maternal stress, which can be exacerbated by inadequate nutrition, has been shown to dampen brain responses to both positive and negative infant cues. This dampened response may hinder a mother’s ability to effectively process and respond to her child’s needs, potentially impacting the child’s emotional development [26]. The interplay between maternal nutrition, stress, and brain activity suggests that ensuring adequate nutritional intake during pregnancy is vital for fostering a nurturing and responsive caregiving environment.

Moreover, the development of neural pathways related to stress, anxiety, and depression is influenced by maternal nutrition. Studies have shown that specific regions of the brain implicated in these pathways are affected by the timing and quality of nutritional intake during pregnancy. For example, equal sampling of younger fetuses could refine our understanding of how the timing of nutritional exposure affects cortical morphology, further emphasizing the importance of maternal diet in prenatal brain development [29].

In summary, maternal nutrition is a key factor in the prenatal development of the emotional brain. Adequate nutritional intake supports the proper development of brain structures involved in emotional regulation and reactivity, while poor nutrition can lead to alterations in brain activity and morphology that may have long-term implications for a child’s emotional health. Ensuring that expectant mothers receive proper nutrition is essential for fostering healthy brain development and emotional well-being in their children.

### 4.6. Hormonal Influences

Hormonal influences play a significant role in the prenatal development of the emotional brain. During pregnancy, maternal hormones such as cortisol, estrogen, and progesterone can cross the placenta and affect fetal brain development. Elevated levels of maternal cortisol, often associated with stress, have been linked to alterations in the fetal brain’s structure and function, particularly in regions involved in emotional regulation such as the amygdala and prefrontal cortex [29]. These hormonal changes can predispose the infant to heightened emotional reactivity and difficulties in emotion regulation later in life. The hypothalamic–pituitary–adrenal (HPA) axis is a critical pathway through which maternal stress hormones influence fetal brain development. The HPA axis regulates the production of cortisol, which can impact the development of neural circuits involved in stress response and emotional regulation.

Studies have shown that maternal stress during pregnancy can lead to increased cortisol levels, which, in turn, can affect the connectivity and functionality of the infant’s brain regions responsible for emotional processing [26]. This suggests that maternal stress and the associated hormonal milieu can have long-lasting effects on the child’s emotional health.

Furthermore, the balance of maternal hormones such as estrogen and progesterone is crucial for maintaining a healthy pregnancy and supporting fetal brain development. These hormones not only support the physical growth of the fetus but also play a role in the maturation of neural circuits involved in emotional regulation. Disruptions in the levels of these hormones, whether due to stress, medical conditions, or other factors, can lead to atypical development of the emotional brain. This highlights the importance of a stable hormonal environment for optimal fetal brain development.

In addition to cortisol, other maternal hormones such as oxytocin have been shown to influence fetal brain development. Oxytocin, often referred to as the “love hormone,” is involved in social bonding and emotional regulation. Higher levels of maternal oxytocin during pregnancy have been associated with better emotional outcomes in children, suggesting that this hormone plays a protective role in the development of the emotional brain [34]. This underscores the complex interplay between various maternal hormones and their collective impact on the child’s emotional development.

The influence of maternal hormones on fetal brain development is further complicated by the timing and duration of exposure. Critical periods of brain development, such as the third trimester of gestation, are particularly sensitive to hormonal influences. During this time, the brain undergoes rapid growth and differentiation, making it more susceptible to the effects of maternal hormones [46]. This period is crucial for the development of brain regions involved in sensory processing, social cognition, and emotional regulation [40]. Therefore, any disruptions in the hormonal environment during this critical window can have profound and lasting effects on the child’s emotional health.

Moreover, the interaction between maternal hormones and genetic factors also plays a role in shaping the emotional brain. Genetic predispositions can influence how the fetal brain responds to maternal hormones, leading to individual differences in emotional development. For instance, certain genetic variants may make some fetuses more sensitive to the effects of maternal cortisol, resulting in greater vulnerability to stress-related emotional disorders [46]. This highlights the need for a comprehensive understanding of both genetic and hormonal influences on fetal brain development.

In summary, maternal hormones significantly influence the prenatal development of the emotional brain. Elevated levels of stress hormones such as cortisol can alter the development of neural circuits involved in emotional regulation, while hormones like oxytocin can have protective effects. The timing and duration of hormonal exposure, as well as genetic factors, further modulate these effects. Understanding these complex interactions is crucial for developing interventions to support optimal emotional development in children.

## 5. Neonatal Period and Early Infancy

### 5.1. Brain Growth and Maturation

Brain growth and maturation during the neonatal period and early infancy are characterized by significant structural and functional changes. These changes are influenced by both genetic and environmental factors, which together shape the development of brain regions associated with emotion.

The amygdala undergoes substantial development during the fetal stage. This includes the establishment of major connections, primarily fronto-limbic, and efferent projections to subcortical regions. Myelination and other maturation processes, such as apoptosis, further modify the amygdala in the late fetal stage [47]. These early structural changes are foundational for the brain’s ability to process and regulate emotions.

Maternal mental health has a profound impact on fetal brain development. Studies have shown that region-dependent cortical alterations related to maternal mental health can be detected in utero [29]. This suggests that the prenatal environment plays a crucial role in shaping the brain’s structural and functional trajectories, potentially influencing emotional development from a very early stage.

The connectivity patterns of the infant brain also provide insights into the neural underpinnings of emotional development. For instance, the association between maternal childhood emotional neglect and infant fronto-amygdala connectivity highlights the long-term impact of maternal experiences on the infant’s brain [34]. These connectivity patterns are promising tools for understanding the risk transmission of neuropsychiatric illnesses.

Early adverse exposures, such as deprivation of emotional input, can lead to accelerated maturation in the amygdala–prefrontal circuitry. This accelerated maturation is thought to occur because young organisms rely on their caregivers to regulate their emotional responses to everyday stressors [48]. The mixed findings regarding the associations of deprivation and threat with accelerated maturation may be due to the differential impact on various mechanisms of neuroplasticity. The processing, interpretation, and expression of negative affect involve multiple brain regions and networks. However, additional research is needed to explore the full extent of how maternal perinatal stress trajectories relate to the structure and function of the neonatal brain [33]. Understanding these relationships is crucial for identifying potential interventions that can support healthy emotional development.

Furthermore, the interplay between anxiety-driven fetal–neonatal neurodevelopmental features and characteristics of the home environment, including parent–infant interactions, is essential for predicting long-term cognitive and behavioral trajectories. Thorough investigations in this area will help determine how postnatal interventions can redirect neurodevelopmental trajectories [32].

The integration of neurobiological measures into the evaluation of support programs across the perinatal period is necessary to fully understand their benefits for children. Elevated maternal stress during pregnancy has been found to affect infant brain development, leading to a higher risk for mental health problems in offspring [24]. This underscores the importance of providing nurturing and responsive caregiving to support optimal brain growth and emotional health.

In summary, brain growth and maturation during the neonatal period and early infancy are influenced by a complex interplay of genetic and environmental factors. Early experiences, particularly those related to maternal mental health and caregiving, play a crucial role in shaping the development of brain regions associated with emotion. Understanding these processes is essential for developing interventions that support healthy emotional development in children.

### 5.2. Development of Emotional Circuits

#### 5.2.1. Right Hemisphere Development in Infancy and Emotional Regulation

The development of the right hemisphere in infancy plays a crucial role in emotional regulation and attachment, influencing how infants cope with stress and navigate their social environments. The right hemisphere of the brain is integral to processing emotional information, regulating stress responses, and facilitating attachment relationships. It develops early in infancy and is closely linked to the limbic system, which is responsible for emotional responses. This area of the brain is dominant in managing the human stress response and is essential for adaptive coping strategies during human interactions. The development of the right hemisphere is associated with the infant’s ability to form secure attachments, which are foundational for optimal emotional health [33].

Traumatic attachments can disrupt and impair the right hemisphere’s regulatory functions, leading to maladaptive emotional responses, and can negatively affecting both infant and adult mental health. Severe attachment failures correlate with inefficient right brain functions, contributing to challenges in emotional regulation [33].

Infants exhibit different emotional regulation strategies as they grow. For instance, younger infants (around 6 months) tend to use gaze aversion and fussing more frequently, while older infants (18 months) begin to engage in more complex strategies like self-soothing and actively directing interactions. This evolution in strategy usage highlights the developmental changes in emotional regulation capabilities, which are influenced by the maturation of the right hemisphere.

The development of the right hemisphere during infancy is critical for emotional regulation. Secure attachments foster effective coping mechanisms, while traumatic experiences can hinder this development, leading to maladaptive emotional responses. Understanding these dynamics is essential for promoting healthy emotional development in infants and addressing potential risks associated with insecure attachments.

#### 5.2.2. Development of the Insula for Emotional Control

The insula is a crucial brain region involved in processing emotions and integrating various cognitive functions. Its development and connectivity play significant roles in emotional regulation and perception. The insula undergoes significant developmental changes from adolescence to adulthood.

A study utilizing high angular resolution diffusion imaging found that the density of fiber connections between the insula and the frontal and parietal cortex decreases with age, while connections to the temporal cortex generally increase [41].

Additionally, developmental trajectories may differ between males and females, particularly in the connectivity between the left insula and the left precentral gyrus. These findings highlight the insula’s complexity and its changing role in emotional and cognitive processing as individuals age [41].

The insula is integral to integrating emotional, cognitive, and sensory–motor systems. It acts as a hub for moderating various functions, including social cognition, empathy, and reward-driven decision-making [42]. Its anterior region is particularly associated with subjective emotional experiences, while the posterior region is more involved in processing sensory information [49].

Overall, the insula develops through a complex interplay of age-related changes and gender differences, significantly impacting emotional processing and regulation. Its connectivity patterns are essential for understanding both typical emotional responses and those altered by psychological conditions. The insights gained from studying the insula could potentially inform therapeutic approaches for emotional dysregulation in various populations.

#### 5.2.3. Amygdala Development

Amygdala development is a critical aspect of the maturation of emotional circuits during the neonatal period and early infancy. The amygdala, a key brain region involved in processing emotions, undergoes significant changes from fetal development through the age of two. These changes are influenced by both genetic and environmental factors, which together shape the emotional health of the child.

Research indicates that children exposed to maternal distress and deprivation exhibit adult-like limbic brain features, such as larger amygdala volumes and functional connectivity patterns typically observed in adults [50]. This suggests that early-life stress can accelerate the maturation of the amygdala, potentially leading to long-term implications for emotional regulation and mental health.

Furthermore, the connectivity between the amygdala and other brain regions, such as the ventro-medial prefrontal cortex, is influenced by maternal factors. For instance, infants of mothers with certain stress trajectories show stronger amygdala connectivity to the ventromedial prefrontal cortex, highlighting the role of maternal stress in shaping the neural circuits involved in emotion regulation [33].

The balance of activation in the anterior cingulate cortex, which is involved in regulatory processes, also plays a role in the development of the amygdala. Differences in child age and temperament are related to variations in dorsal and ventral anterior cingulate cortical activation during regulatory episodes, suggesting that these factors may influence the maturation of the amygdala and its connectivity with other brain regions [41].

Additionally, prenatal maternal depression has been associated with changes in child brain structure, including the amygdala. Studies have found that maternal stress during the second trimester is particularly impactful, emphasizing the need for longitudinal research to further understand the role of prenatal stress on the developmental trajectory of the amygdala and other related brain regions [21]. Sex differences in amygdala development have also been examined, with some studies reporting no significant differences in prenatal exposures and early brain development. However, the functional connectivity between the amygdala and prefrontal areas is already established in late gestation, indicating that these connections are crucial for early emotional development [46].

The lack of legislated temporal protection for the establishment of a nurturing maternal-infant relationship can have long-term effects on the emotional health of children, particularly males. Males, whose right brains mature more slowly than females, are more vulnerable to early relational stressors, which can impact the development of the amygdala and increase susceptibility to developmental disorders and externalizing psychopathologies [40].

Overall, the development of the amygdala is a complex process influenced by a combination of genetic, environmental, and maternal factors. Understanding these influences is essential for promoting healthy emotional development and mitigating the risks associated with early-life stress and maternal distress.

#### 5.2.4. Ventral Striatum Development

The ventral striatum, a critical component of the brain’s reward system, undergoes significant development during the neonatal period and early infancy. This region is integral to processing rewards and is closely linked to emotional and motivational behaviors. The maturation of the ventral striatum is influenced by both genetic and environmental factors, which together shape its structural and functional properties.

During fetal development, the ventral striatum begins to form connections with other brain regions, including the prefrontal cortex and the amygdala. These connections are essential for the integration of emotional and cognitive processes. Studies have shown that early developmental stress can impact the structural organization of these circuits. For instance, children exposed to poverty and maltreatment exhibit altered amygdala volume and premature myelination of amygdala circuits, which can affect the ventral striatum’s development [51].

The autonomic nervous system (ANS) also plays a role in the development of the ventral striatum. The ANS, which supports mammalian social behavior, starts to develop during the last trimester of fetal life. A right-lateralized circuit of emotion regulation, which supports the functional dominance of the right side of the brain in regulating autonomic function, is proposed to be crucial for the development of the ventral striatum [36].

Prenatal hemispheric differences in the functional and structural connectome are also relevant. Research using resting-state functional MRI (rs-fMRI) has found that the ventral frontal-temporal cortex module becomes more left-lateralized in older fetuses, potentially developing into Broca’s and Wernicke’s Areas. This lateralization may influence the development of the ventral striatum and its connections with other brain regions [30].

Emotional neglect during a mother’s own childhood has been shown to affect the connectivity between the amygdala and the ventral vmPFC in neonates. This effect is significant even after controlling for maternal prenatal distress, indicating that early-life experiences of the mother can have a lasting impact on the child’s brain development, including the ventral striatum [31].

The development of the ventral striatum is also linked to the differentiation of the amygdala. During the fetal period, the differentiation of the main nuclei of the amygdala is completed, and further development causes a change in the position, or a rotation, of structures around the medial eminence. This process is unique to humans and highlights the complexity of the ventral striatum’s development [47].

Longitudinal studies are essential to clarify the directionality of associations between stress exposure, maternal mood, and brain responses during pregnancy and the early postpartum period. Understanding these relationships can provide insights into how stress impacts the maternal brain and, consequently, the development of the ventral striatum in infants [26].

In summary, the development of the ventral striatum is a complex process influenced by a combination of genetic, environmental, and experiential factors. Early developmental stress, maternal experiences, and the autonomic nervous system all play crucial roles in shaping the structural and functional properties of this critical brain region. Further research is needed to fully understand the mechanisms underlying these influences and their long-term implications for emotional and motivational behaviors.

#### 5.2.5. Connectivity Between Subcortical and Cortical Areas

Connectivity between subcortical and cortical areas plays a crucial role in the development of emotional circuits during the neonatal period and early infancy. The maturation of these connections is essential for the regulation and processing of emotions, which are foundational for later emotional health and behavior.

The amygdala, a key subcortical structure involved in emotional processing, shows significant connectivity with various cortical regions. This connectivity is not static but undergoes dynamic changes during early development. For instance, maternal stress during pregnancy has been shown to influence the functional connectivity of the neonatal amygdala. However, it is the trajectory of maternal stress over time, rather than stress at a single time point, that is predictive of these connectivity patterns [33]. This suggests that the cumulative exposure to stressors during pregnancy can have a lasting impact on the brain’s emotional circuits.

Postmortem studies have provided evidence for hierarchical developmental changes in the brain, highlighting the selective stabilization and regional elimination of excitatory synaptic connections. These processes occur first in the sensorimotor cortex and later in the prefrontal cortex, which is crucial for higher-order cognitive functions. Such changes are believed to alter the balance between excitatory and inhibitory neural connections within these regions, thereby influencing emotional regulation [52]. The ability to predict typical and atypical patterns of emotional development is essential for early intervention. For example, understanding the connectivity patterns between the amygdala and cortical areas can help identify children at risk for emotional disorders. Preventative therapies, such as emotion regulation training, can then be targeted to enhance mental health outcomes [10]. This approach underscores the importance of early detection and intervention in promoting emotional well-being.

Novel approaches are needed to characterize the functional development of brain regions involved in emotion. Traditional methods like seed-based approaches or independent component analysis may not be sufficient. Instead, advanced techniques that can create detailed “heatmaps” of brain activity are required to better understand these developmental processes [38]. Such techniques can provide a more comprehensive view of how subcortical-cortical connectivity evolves and its implications for emotional development. Research has also shown that the development of connections between cortical regions and the amygdala is significantly correlated with gestational age. This indicates that the timing of these connections is critical for proper emotional development. For example, specific connections between cortical parcels and the amygdala develop in a gestational age-dependent manner, highlighting the importance of prenatal factors in shaping these neural circuits [30].

**Figure 3:**
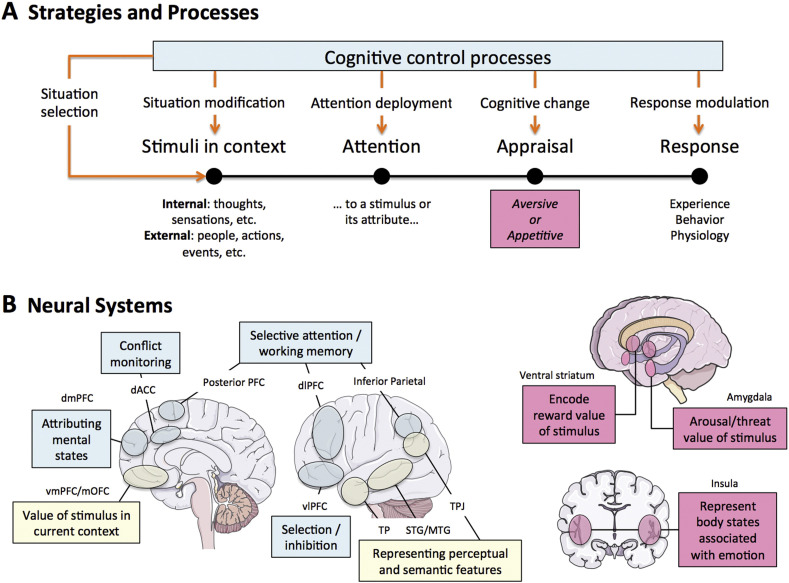
A model of the cognitive control of emotion (MCCE). (**A**) A diagram showing the steps in the processing of emotions and how they can be controlled by cognitive control processes (blue box). The stages of the emotion-generating sequence that are influenced by various emotion regulation techniques can be used to understand their effects (the red arrows descending from the box representing cognitive control processes). The pink box at the assessment step is supposed to show that this process is supported by the brain systems that produce emotion. (**B**) Neural systems that generate such reactions (left, pink boxes) use cognitive methods, like reappraisal, to regulate emotion (left, blue boxes), and play an unclear or mediating function in reappraisal (left, yellow boxes).

Furthermore, studies have demonstrated that the quality of the postnatal environment can confound the association between prenatal factors and brain development. By focusing on neonates, researchers can limit the influence of postnatal factors and gain a clearer understanding of how prenatal environments impact brain structure and function. This approach has been used to measure hippocampal and amygdala structures in neonates, providing insights into the early development of emotional circuits [53]. When all of the relevant systems are developing and integrated by the end of a child’s second year postpartum, the schematic represented in Figure 3 illustrates the anatomical connectivities of integrated emotional functioning.

In summary, the connectivity between subcortical and cortical areas is a dynamic and critical aspect of emotional development during the neonatal period and early infancy. The interplay between prenatal and postnatal factors, the timing of neural connections, and the use of advanced imaging techniques all contribute to our understanding of how these emotional circuits develop and function. This knowledge is essential for identifying at-risk children and implementing early interventions to promote emotional health.

### 5.3. Role of Early Experiences

#### 5.3.1. Attachment and Bonding

Attachment and bonding during the neonatal period and early infancy are critical for the emotional and psychological development of a child. The interactions between a mother and her infant, characterized by emotional exchanges and synchrony, play a significant role in shaping the infant’s mental health. Stern’s observations [54] highlight that the dynamic nature of mother–infant emotional exchanges, where patterns of engagement vary across different dyads, strongly influences the infant’s mental health. These affective interactions are essential for the development of secure attachment, which is foundational for the child’s future emotional regulation and social functioning.

The quality of caregiving, particularly the sensitivity and responsiveness of the mother, is crucial in this context. Belsky and Fearon [55] observed that children with secure attachment histories whose mothers became insensitive during toddlerhood exhibited lower psychosocial functioning scores at three years of age. This finding underscores the importance of consistent and sensitive caregiving in promoting positive child outcomes, even in the presence of early insecure attachment histories [1]. The ability of a caregiver to attune to and regulate the infant’s internal states through nonverbal affective communications, as proposed by Schore [40], is a key aspect of this process. These right-brain-to-right-brain interactions facilitate the regulation of the infant’s autonomic and central nervous system arousal, contributing to the child’s emotional stability.

Furthermore, the emotional neglect experienced by the mother during her own childhood can have distinct and separate effects on the child’s neurodevelopment, independent of prenatal distress. This suggests that the emotional environment provided by the caregiver, shaped by her own experiences, can significantly impact the child’s emotional development [34]. The vulnerability of the fetal brain, particularly the limbic areas such as the amygdala, hippocampus, and hypothalamus, to environmental influences like maternal anxiety, further emphasizes the importance of a nurturing and supportive caregiving environment [9,32,56].

Recent studies highlight that parent–infant synchronization—defined as the mutual coordination of rhythms, affective expressions, and vocal tone—has a profound impact on the development of neural connectivity within emotional circuits, particularly between the prefrontal cortex and the amygdala. This connectivity forms a key neurobiological foundation for emotional regulation, stress response, and affective learning. For instance, Feldman [57] demonstrated that synchronous interactions during the first year of life promote the formation of fronto-limbic neural pathways. Pratt et al. [58] found that infants who experienced high levels of parental synchrony showed increased amygdala–prefrontal connectivity, corresponding with the caregiver’s emotional responsiveness. Similarly, Kim et al. [26] showed that strong affective synchrony serves as an experiential mechanism guiding the development of neural pathways involved in emotional processing. These findings underscore the critical role of early social experiences in shaping both the structure and function of the developing brain.

The synchronization of emotional exchanges between the mother and infant, often mediated by facial expressions, posture, and tone of voice, is a critical component of attachment and bonding. Trevarthen’s research on developmental brain laterality indicates that the prosody of the mother’s voice is particularly influential, as it is processed by the infant’s right hemisphere, which is involved in emotional processing [40]. This highlights the importance of the quality of maternal communication in fostering secure attachment and emotional development.

In summary, the early experiences of attachment and bonding, mediated by sensitive and responsive caregiving, are fundamental to the emotional and psychological development of the child. These interactions not only influence the immediate emotional well-being of the infant but also have long-term implications for their social and emotional functioning. The interplay between the caregiver’s own emotional history and the quality of caregiving provided underscores the complex nature of attachment and bonding during this critical period.

#### 5.3.2. Sensory Experiences

Sensory experiences play a crucial role in the maturation of brain regions associated with emotion from fetal development through early infancy. The brain’s plasticity during this period allows it to adapt and reorganize in response to various sensory inputs, which can significantly influence emotional development.

During fetal development, the brain begins to form the foundational structures necessary for processing sensory information. Maternal psychological stress during pregnancy has been shown to affect the development of these structures, particularly the amygdala, which is involved in both stress reactivity and reward processing [43]. Increased maternal stress can lead to alterations in the amygdala’s developmental trajectory, potentially affecting the infant’s emotional regulation capabilities. In the neonatal period, sensory experiences continue to shape the brain’s development. For instance, increased fetal cortical gyrification and sulcal depth, which are influenced by maternal psychological distress, have been associated with decreased social–emotional competence in infants [24]. This suggests that early sensory experiences, even those occurring in utero, can have lasting effects on emotional development.

The postnatal environment further contributes to the maturation of brain regions involved in emotion. New mothers exhibit enhanced neural activation in the emotion regulation and cognitive control circuits, including the anterior cingulate cortex and the PFC. These regions are crucial for effective emotion regulation, especially in the context of distressful interactions with their infants. The PFC’s strong functional connectivity to the amygdala allows it to downregulate amygdala activation in response to threats and negative stimuli, thereby supporting sensitive parenting behaviors. Moreover, the quality of caregiving plays a significant role in shaping an infant’s emotional health.

Responsive and nurturing caregiving can promote the development of efficient cortical networks, leading to better emotional regulation and social–emotional competence. Conversely, negative childhood experiences, such as emotional neglect, can result in increased distress responses in mothers, which may affect their ability to respond sensitively to their infants’ cues [26]. The importance of early sensory experiences is further highlighted by the observation that certain brain systems may mature more quickly in environments that provide frequent and varied sensory inputs. For example, repetitive use of language systems in high-SES environments can lead to stronger connections between language-processing regions [17]. This enhanced connectivity supports the development of more efficient neural networks, which are essential for emotional and cognitive functions.

In summary, sensory experiences from fetal development through early infancy play a critical role in the maturation of brain regions associated with emotion. These experiences, influenced by both prenatal and postnatal environments, shape the brain’s structural and functional development, ultimately impacting emotional health and social–emotional competence. Understanding the interplay between sensory experiences and brain development can inform interventions aimed at promoting optimal emotional development in early childhood.

#### 5.3.3. Early Social Interactions

Early social interactions play a crucial role in the emotional and cognitive development of infants. These interactions are foundational for the establishment of effective emotion regulation and social–emotional processing capabilities. The capacity for emotion regulation is often rooted in the early interactions between the parent and the infant, which are essential for the development of social and emotional competencies [1].

During the neonatal period and early infancy, the brain undergoes significant maturation, particularly in regions associated with emotion and social interaction. The right brain, which develops earlier than the left, is heavily involved in these processes. This early development is critical as it sets the stage for the infant’s ability to engage in intersubjective experiences, which are nonverbal and involve the mutual regulation of emotions between the infant and the caregiver.

The functional architecture of the brain, including the Default Mode Network (DMN), is associated with emotional functioning and is influenced by both genetic factors and prenatal experiences [51]. These early brain connectivity metrics are crucial as they may shape neurodevelopment and impact the infant’s ability to process and respond to emotional stimuli. For instance, fMRI studies have shown that infants as young as from 3 to 7 months old exhibit increased hemodynamic activity in brain regions such as the orbitofrontal cortex (OFC) and insula in response to sad vocalizations, indicating the early differentiation of emotional stimuli [52].

Moreover, the social brain areas, including the amygdala, anterior cingulate, insula, and medial orbitofrontal cortex, are mapped out in children and show variability with age, which may underlie individual differences in social–emotional processing [18]. These regions are integral to the successful establishment of social interaction routines and emotional regulation capabilities, which are fundamental for learning and the emergence of other cognitive and executive functions [38].

The early interactions between the infant and the caregiver are not only crucial for immediate emotional regulation but also for the long-term development of secure attachment bonds. These bonds are formed through interactively regulated affect transactions that maximize positive and minimize negative affect, thereby creating a secure attachment between the mother and the infant [40]. This secure attachment is essential for the infant’s ability to self-regulate and interactively regulate emotions, which are critical for adaptive functioning throughout life.

In summary, early social interactions are vital for the emotional and cognitive development of infants. These interactions influence the maturation of brain regions associated with emotion and social processing, shape neurodevelopment, and contribute to the formation of secure attachment bonds. The nurturing and responsive caregiving during this critical period is essential for the child’s emotional health and overall development.

## 6. Infancy to Toddlerhood

### 6.1. Continued Brain Maturation

Continued brain maturation during infancy to toddlerhood is a complex and dynamic process influenced by various factors, including prenatal conditions, early experiences, and environmental influences. The development of the brain regions associated with emotion, such as the hippocampus, amygdala, and neocortex, is particularly critical during this period.

Prenatal maternal psychological distress has been shown to impact fetal brain development, specifically affecting the cortical gyrification index and sulcal depth. These disturbances in cerebral cortical folding can mediate the association between prenatal maternal distress and later neurodevelopmental problems in infancy. The hippocampus, a key brain region involved in emotion regulation and cognitive functions, is notably affected by prenatal stress, which can lead to adverse socio-cognitive outcomes in infants [16].

The development of cortical representations of behavioral actions, mediated by frontal regions, is influenced by an infant’s experiences, particularly those involving goal-directed behavior and emotional regulation [19]. This highlights the importance of early experiences in shaping the brain’s emotional and cognitive development.

Attention to faces at 7 months of age does not predict emotion understanding or mentalizing abilities at 48 months, suggesting that other factors play a more significant role in the development of these skills [23]. However, increased attention to faces during infancy is associated with better emotion regulation abilities later in life, indicating the importance of early social interactions in emotional development.

The gestational environment significantly influences brain development, with adverse prenatal factors inducing various brain alterations. These changes can have long-lasting effects on physiology, behavior, and cognition during adulthood [59]. Key brain regions, such as the hippocampus, amygdala, and neocortex, are particularly susceptible to these influences, which can impact emotional regulation and cognitive functions.

Alterations in brain regions during pregnancy and the postpartum period can lead to difficulties in regulating negative emotions, such as elevated anxiety and depressive mood [26]. The regulation of the HPA axis undergoes drastic changes during this time to support pregnancy and parenting, further influencing emotional development.

Caregiving responsiveness, maternal mental health, couple relationships, and socioeconomic status are critical factors influencing infant behavioral regulation and attachment status between 12 and 18 months. Perinatal factors most proximal to the infant have the strongest associations with social–emotional status, emphasizing the importance of a supportive and nurturing environment [39].

The neurodevelopment of emotion regulation involves changes and interactions across cognitive and emotional development. Brain network activity metrics can explain a significant portion of the variance in emotion regulation ability, even after accounting for age [60]. This finding underscores the importance of understanding how brain networks evolve and interact during early development.

Interactions with the infant induce structural changes in the mother’s brain, particularly in regions associated with motivation and sensitivity to infant cues. These changes are expressed in functional increases in maternal motivation and sensitivity, which are crucial for the infant’s emotional development [36].

In summary, the continued maturation of brain regions associated with emotion from fetal development through the age of two is a multifaceted process influenced by prenatal conditions, early experiences, and environmental factors. Nurturing and responsive caregiving plays a vital role in shaping a child’s emotional health, highlighting the need for early interventions and supportive environments to promote optimal development.

### 6.2. Development of Self-Regulation

#### 6.2.1. Emotional Regulation Strategies

Emotional regulation strategies during infancy and toddlerhood are crucial for the development of self-regulation. The maturation of brain regions associated with emotion, such as the prefrontal cortex and amygdala, plays a significant role in this process. The prefrontal cortex is essential for executive functions, which include the ability to regulate attention and emotion, set goals, plan, and organize [31]. These functions are foundational for learning and problem-solving, and their development is largely complete by the age of five. The interaction between the prefrontal cortex and the amygdala is particularly important for emotional regulation. Studies have shown that prenatal distress can affect the integrity of the amygdala–prefrontal circuits, which are critical for emotion regulation [21]. This suggests that early experiences, even before birth, can have lasting impacts on a child’s ability to manage emotions.

Breastfeeding has been linked to better emotion regulation in infants, as it provides not only nutritional benefits but also regular maternal physical touch and attunement to the infant’s needs. This interaction promotes the development of the prefrontal cortex, which is involved in attachment and emotional regulation. McIntosh et al. [39] indicate that nutrition and maternal interaction during feeding are crucial for the emotional development of infants.

Furthermore, the maturation of the frontal lobe enhances a child’s ability to control their emotions and exercise inhibitory control over their thoughts and actions. This development allows children to use verbalizations to achieve self-regulation of their feelings [57]. The ability to verbalize emotions is a significant milestone in emotional regulation, as it provides children with tools to express and manage their feelings effectively.

The role of maternal sensory signals in emotional outcomes has also been studied. Unpredictable maternal sensory signals can relate to internalizing behaviors such as depression and anxiety in children. This highlights the importance of consistent and responsive caregiving in shaping a child’s emotional health. The findings from [61] emphasize that early-life experiences with caregivers can influence emotional outcomes significantly.

Additionally, the socioemotional orienting between a mother and infant triggers the ascending reticular activating system, which involves neurochemical and circulatory signaling that produces body-wide physiological responses. This mechanism is separate from the thalamo-cortico-amygdaloid pathway and underscores the complexity of emotional regulation processes. Xiong et al. [62] propose that the interaction between mother and infant has profound effects on the infant’s emotional regulation through these physiological mechanisms.

In summary, the development of emotional regulation strategies in infants and toddlers is influenced by the maturation of brain regions, early experiences, and environmental factors. Nurturing and responsive caregiving, including breastfeeding and consistent maternal sensory signals, play a crucial role in shaping a child’s emotional health. The interplay between the prefrontal cortex and amygdala, along with other neural circuits, underpins the ability to regulate emotions effectively during this critical period of development.

#### 6.2.2. Role of Caregivers

The role of caregivers in the development of self-regulation during infancy to toddlerhood is crucial. Early caregiving experiences significantly influence the maturation of brain regions associated with emotion regulation. The anterior insula, a key region involved in emotional empathy, plays a vital role in a mother’s ability to share and respond to her child’s distress. Increased amygdala response to infant cues can be linked to sensitive parenting, but it can also be associated with stress reactivity and intrusive parenting depending on the context and its connectivity to other brain regions [26].

The development of emotion regulatory abilities expands dramatically during the second half of the first year of life. This period is marked by significant changes in the frontal region of the brain, which is responsible for directed and sustained attention. Studies of brain-damaged adults have shown that lesions in the frontal lobe result in distractibility and difficulties in monitoring temporal sequences, suggesting that the frontal region is crucial for these regulatory skills [19].

Early parenting practices can alter the emotion circuitry in the brain. The regression model used in studies includes various explanatory variables such as motion parameters and stimulus types (fear and neutral), indicating that different types of stimuli can affect emotional responses and regulation [63]. The presence of nurturing and responsive caregiving is essential for the healthy development of these brain regions.

The postnatal environment, including caregiving behavior and the presence of siblings, plays a critical role in the development of functional connectivity and negative affectivity. Examining the transactional associations helps elucidate the pathways implicated in early emotional development. The early postnatal environment, as outlined by bioecological models, is embedded in multiple layers of environmental influences, both proximal and distal to the child’s immediate surroundings. These influences can significantly impact the development of the fronto-limbic structure and function, which are associated with an increased risk for later emotional and behavioral problems [24].

Furthermore, the connectivity between the amygdala and the DMN in newborns has been positively associated with parent-reported fearful behaviors at six months of age. This highlights the vulnerability of early infancy to perturbations in brain development, which can have long-term consequences for mental health [51]. Bisiacchi and Cainelli [36] indicate that studying the infant brain proximal to birth minimizes, but does not eliminate, the potential influences of the postnatal environment. A focused investigation of maternal childhood maltreatment effects on prenatal brain development is needed to provide evidence of prenatal programming’s potential.

In summary, the role of caregivers is integral to the development of self-regulation in infants and toddlers. Early experiences and environmental factors, particularly those related to caregiving, have a profound impact on the maturation of brain regions involved in emotion regulation. Nurturing and responsive caregiving practices are essential for fostering healthy emotional development during this critical period.

#### 6.2.3. Impact of Environment

The impact of the environment on the development of self-regulation from infancy to toddlerhood is profound and multifaceted. Early experiences and environmental factors play a crucial role in shaping the emotional and cognitive development of children. The brain regions associated with emotion, such as the amygdala and ventral striatum, undergo significant maturation during this period, and their development is highly sensitive to external influences [52].

During fetal development, the brain undergoes dramatic changes, even within a single week of gestation. The use of age-specific fetal templates has allowed researchers to capture the anatomical variability of the fetal population and assess functional connectivity across gestation [30]. This period is critical as it sets the foundation for future emotional and cognitive development.

Postnatal environmental factors, such as maternal prenatal stress, can moderate the associations between early stress and infant brain development. Protective or promotive postnatal environments can buffer the negative effects of prenatal stress, promoting resilience in the developing brain [24]. The HPA axis plays a significant role in this process, influencing the infant’s ability to regulate stress and emotions.

The transition from natural sleep to awake states, such as watching cartoons, between the ages of two and four years, can influence the observed changes in brain development. Limbic and subcortical regions, which are crucial for emotional processing, show the most significant developmental changes during this period [34]. These changes highlight the importance of considering the child’s state when assessing brain development.

The early postnatal environment, including the quality of caregiving, is essential for the development of self-regulation. Neonates exposed to nurturing and responsive caregiving show better neural connectivity and emotional regulation. Conversely, maternal childhood emotional neglect can negatively impact offspring neural development, as mothers who experienced neglect may engage in fewer behaviors that buffer their neonates from stress [34].

Socioeconomic status and other contextual variables, such as race and ethnicity, also play a crucial role in shaping brain development. These factors influence the exposure–outcome relationships, making it challenging to disentangle the effects of different early exposures and their interactions with the genome [3]. Understanding these complex interactions requires comprehensive research across diverse social and environmental contexts.

The early neural representations of sensory stimuli, such as faces and speech, support socioemotional and language development as infants interact with their caregivers. These early experiences are crucial for the development of abstract categories, which are essential for emotional and cognitive growth [12]. The quality of these interactions can significantly influence the child’s ability to develop self-regulation skills.

In summary, the environment plays a critical role in the development of self-regulation during infancy to toddlerhood. Early experiences, maternal stress, caregiving quality, and socioeconomic factors all contribute to shaping the emotional and cognitive development of children. Understanding these influences is essential for promoting optimal developmental outcomes.

### 6.3. Socioemotional Development

#### 6.3.1. Recognition of Emotions

Recognition of emotions in infants and toddlers is a complex process that involves the maturation of various brain regions and is significantly influenced by early experiences and environmental factors. During the first months of life, the posterior parietal cortex, which is associated with somatosensory processing, matures due to high levels of tactile stimulation from the maternal environment. This tactile input is crucial for the development of the infant’s ability to recognize and respond to emotional cues. fMRI studies have shown that a significant milestone in the development of the infant brain occurs around eight weeks of age, marked by a rapid metabolic change in the primary visual cortex, which is essential for processing visual emotional stimuli [44].

The development of emotion recognition is also closely linked to the formation of subcortical–cortical functional connectivity patterns in the fetus, which are influenced by the maternal psychological context. Anxiety-driven modulations in the maternal environment can impact these connectivity patterns, which, in turn, affect the infant’s ability to recognize and process emotions at birth and during early childhood [32]. This highlights the importance of a nurturing and stable maternal environment for the healthy development of emotional recognition capabilities.

As infants grow, the maturation of neural regions and pathways that underlie working memory and inhibitory control plays a crucial role in the development of emotion regulation and recognition. By around the age of four, improvements in inhibitory control and metacognitive abilities are observed, which are associated with the maturation of the prefrontal cortex. This region is responsible for executive functions that are essential for emotion regulation and recognition [64]. The ability to recognize and regulate emotions is thus deeply intertwined with the development of cognitive control mechanisms.

Social–emotional development in infants and toddlers is further influenced by early interactions with caregivers. Attachment theory posits that the formation of secure attachment relationships is a fundamental organizing force in infant social development. These early interactions not only promote survival but also form the basis for more complex representations of caregivers as available and responsive, which are crucial for the development of emotion recognition skills [1]. The quality of caregiving, therefore, plays a significant role in shaping an infant’s ability to recognize and respond to emotional cues.

Research has also shown that experiences of deprivation during infancy and toddlerhood can have a profound impact on brain and behavioral outcomes. Critical periods of development, such as those for language and attachment, are times when the absence of necessary environmental input can lead to permanent deficits in specific functions supported by particular groups of neurons and synaptic connections [48]. This underscores the importance of providing a stimulating and responsive environment during these sensitive periods to support the development of emotion recognition abilities.

Longitudinal studies are essential for understanding how the trajectory of brain network activity changes over time and relates to the development of emotion regulation and recognition skills. Cross-sectional research provides a starting point, but longitudinal data can offer deeper insights into the dynamic processes involved in emotional development [52]. This approach can help identify critical periods and factors that influence the maturation of brain regions associated with emotion recognition. In summary, the recognition of emotions in infants and toddlers is a multifaceted process that depends on the maturation of specific brain regions, early experiences, and the quality of caregiving. The development of functional connectivity patterns, the maturation of cognitive control mechanisms, and the formation of secure attachment relationships all contribute to the infant’s ability to recognize and respond to emotional cues. Providing a nurturing and responsive environment during critical periods of development is essential for fostering healthy emotional development.

#### 6.3.2. Expression of Emotions

The expression of emotions during infancy to toddlerhood is a complex process influenced by both neurodevelopmental and environmental factors. The maturation of brain regions associated with emotion begins in the fetal stage and continues through the age of two, with significant changes occurring in the structure and function of these regions. Early experiences play a crucial role in shaping emotional development. For instance, maternal psychological distress has been linked to specific fetal MRI-based brain measures, including volumetric growth of the hippocampus and cortical folding metrics, which are critical for emotional processing [16]. These early influences underscore the importance of a nurturing and responsive caregiving environment. The development of emotion regulation strategies is another key aspect of emotional expression.

Functional brain imaging research has shown that different strategies, such as expressive suppression and cognitive reappraisal, are employed to manage emotional experiences. These strategies involve distinct neural mechanisms and are crucial for adaptive emotional responses [64]. The ability to regulate emotions effectively is associated with the maturation of neural circuits, particularly those involving the amygdala and ventral striatum, which are implicated in emotional reactivity and regulation.

The intersubjective mechanism of human development, which involves the earliest relationship between the infant and the mother, is fundamental to emotional expression. This relationship is characterized by energy shifts and rapid affective changes, which are experienced as emotional responses [34]. The quality of this early relationship can significantly impact the child’s emotional health and development.

Neuroimaging findings suggest a dynamic and hierarchical course of development in neural circuits related to emotion. Changes in subcortical regions are associated with emotional reactivity, while cortico-subcortical changes are implicated in emotion regulation. Eventually, cortico-cortical changes support the regulation of emotionally driven attention and reappraisal [52]. These developmental trajectories highlight the importance of early interventions to support healthy emotional development. Individual differences in emotion regulation are often related to variations in the caregiving context.

Children with different temperaments face unique challenges in regulating their emotions. For example, a child with a positive disposition and a high threshold for distress will have different regulatory needs compared to a child prone to intense and persistent negative emotions [1]. This variability underscores the need for tailored approaches to support each child’s emotional development.

In summary, the expression of emotions during infancy to toddlerhood is shaped by a combination of neurodevelopmental changes and early environmental influences. Nurturing and responsive caregiving is essential for fostering healthy emotional development, as it supports the maturation of neural circuits involved in emotion regulation and reactivity. Understanding these processes can inform strategies to promote emotional well-being in young children.

#### 6.3.3. Development of Empathy

The development of empathy is a multifaceted process that begins early in life and is influenced by various factors, including brain maturation, early experiences, and environmental conditions. Empathy, the ability to understand and share the feelings of another, is crucial for socioemotional development and is underpinned by complex neural mechanisms.

The neural basis of empathy involves several brain regions, including the medial and ventromedial prefrontal cortex, the temporoparietal junction, and the prefrontal cortex. These areas are responsible for self- and other-awareness, perspective-taking, emotion regulation, and the appraisal of social contexts [57]. The development of these brain regions is critical for the emergence of empathetic behaviors.

Empathy development is also closely linked to the quality of caregiving. Responsive and nurturing caregiving can significantly influence the neural circuits involved in emotion regulation and empathy. For instance, the presence of maternal stress during pregnancy has been shown to affect the emotional development of infants, potentially leading to increased negative emotionality [33]. This underscores the importance of a supportive and stress-free environment for the developing child.

Furthermore, the interaction between the caregiver and the child plays a crucial role in shaping the child’s emotional health. The concept of intersubjectivity, which involves rapid emotional communications between the minds of both members of the therapeutic alliance, is essential in this context. This nonverbal communication allows the child to make sense of another’s mind, facilitating the development of empathy [40].

The impact of early experiences on empathy development is also evident in the context of the COVID-19 pandemic. Prenatal and postnatal maternal distress during the pandemic has been associated with slower infant socioemotional development. Factors such as mask-wearing, which obstructs face recognition, may further affect brain development and the ability to empathize [60]. Longitudinal studies are needed to determine whether these socioemotional and cognitive delays are transient or long-lasting. Empirical evidence suggests that the development of empathy is a dynamic process that involves the integration of multiple brain regions and is influenced by both biological and environmental factors. The heuristic models of brain development, which often emphasize reward versus control systems, may not fully capture the complexities of emotionally driven behaviors and the development of empathy [52]. A more comprehensive approach that considers the interaction between various neural mechanisms and environmental influences is necessary to understand the development of empathy fully.

In summary, the development of empathy from infancy to toddlerhood is a complex process influenced by brain maturation, early experiences, and environmental factors. Nurturing and responsive caregiving plays a crucial role in shaping a child’s emotional health and the ability to empathize with others. Understanding the neural and environmental underpinnings of empathy development can provide valuable insights into promoting healthy socioemotional development in children.

## 7. Discussion

The maturation of brain regions involved in emotion from fetal development through the age of two represents a complex interplay of genetic, environmental, nutritional, and caregiving factors. This period is marked by significant growth and differentiation in neural circuits crucial for emotional regulation and expression. The processes of neurogenesis and neural migration lay the groundwork for establishing these circuits, while synaptic pruning and myelination further refine them to enhance efficiency.

Early experiences are pivotal in shaping the trajectory of emotional development. Responsive and nurturing caregiving positively impacts neural circuit development, promoting healthy emotional regulation and reducing vulnerability to mental health issues later in life. Conversely, adverse early experiences such as neglect or chronic stress can disrupt neural maturation, leading to difficulties in emotional regulation.

Prenatal factors significantly influence brain development. Elevated levels of placental CRH and maternal stress during pregnancy have been associated with alterations in brain structure and function that affect emotional health. Proper nutritional intake during pregnancy is essential as it supports the development of brain structures involved in emotional regulation and reactivity. Poor nutrition can lead to changes in brain activity and morphology with long-term implications for a child’s emotional health. These findings underscore the importance of prenatal care, maternal well-being, and adequate nutrition for optimal fetal neurodevelopment.

The first years of life are characterized by rapid changes in brain structure and function, driven by both intrinsic genetic programs and extrinsic environmental factors. The frontal lobe, particularly the prefrontal cortex, plays a crucial role in early behavioral development despite earlier assumptions about its functionality during infancy. The development of socioemotional skills underscores the critical nature of early experiences in shaping the social brain.

Research highlights the necessity of early interventions, supportive caregiving practices, and proper maternal nutrition to foster healthy emotional development. Understanding the interplay between genetic predispositions, environmental influences, hormonal impacts, and nutritional factors is essential for developing strategies to mitigate the impact of adverse early experiences.

These insights provide a comprehensive understanding of how early-life stages shape emotional health. They inform future research and interventions aimed at promoting emotional well-being from the earliest stages of life by emphasizing the importance of maternal nutrition alongside other critical factors.

Despite significant advancements in understanding the maturation of brain regions associated with emotion from fetal development through the age of two, several gaps in current knowledge remain. One major gap is the lack of comprehensive longitudinal studies that track the development of amygdala–hippocampal connectivity across early and middle lifespans. This deficiency makes it challenging to interpret deviations from normative development and understand the long-term implications of early-life experiences on emotional health.

Another critical gap is the limited representation of diverse racial and ethnic backgrounds in research studies. This underrepresentation hinders the ability to generalize findings and understand unique and common risk and protective factors for mothers and children from different backgrounds. Greater efforts are needed to include minority populations to ensure that research findings are reflective of the broader population. Furthermore, the current literature on resilience factors is sparse. While some factors, such as appropriate social support and adaptive coping strategies, have been identified, more research is needed to explore other potential resilience factors that could mitigate the adverse effects of early-life stress and environmental influences on emotional development [26].

The impact of prenatal maternal distress on neonatal neural circuit maturation is another area that requires further investigation. Although there is evidence supporting the Developmental Origins of Health and Disease hypothesis, which links prenatal maternal distress to early alterations in neonatal neural circuits, more research is needed to understand the specific mechanisms and long-term outcomes of these early alterations [21].

Additionally, the reversibility of alterations caused by adverse childhood experiences and prolonged chronic stress in adulthood remains unclear. It is essential to determine whether interventions can reverse these alterations and improve emotional health outcomes in affected individuals [42].

The integration of multi-modal data and advances in bioinformatics platforms have enhanced the capabilities for secure data storage, harmonization, and sharing. However, there is still a need for improved communication of detailed research findings, tools for data access and analysis, supporting documentation, and necessary training materials to facilitate the effective use of these data in research [3].

Finally, the normative development of neural architecture relevant to cognitive effort and emotion regulation processes in youth with superior inhibitory control is not well understood. Future research should explore whether stronger baseline expression of these neural architectures is a result of implicit and/or explicit emotion regulation processes and how these processes impact mental resources and development over time [62].

Addressing these gaps in knowledge is crucial for advancing our understanding of the factors that influence emotional development during early childhood and for developing effective interventions to promote emotional health.

Outstanding questions regarding the timing, chronicity, types, and severity of stress exposure, as well as the role of race/ethnicity, are critical for understanding the causal impact of stress on emotional development [23].

Exploring the functional connectivity of the human brain and understanding the complex interactions of molecular, physiological, and neural systems across developmental trajectories are essential scientific building blocks. These efforts are crucial for addressing the large unmet clinical needs related to emotional development. The use of reference models for developing functional connectivity trajectories can help clarify the nature of deviations due to early adversity exposure [42]. This approach is particularly relevant for cross-cultural studies, as it allows for the identification of culture-specific factors that influence emotional development.

Neuroscience offers valuable tools and methodologies for examining the structural and functional changes in the brain. For instance, platforms that house data from MRI, EEG, wearable sensors, and genetics can harmonize with existing large-scale neurodevelopmental research efforts. These platforms enable researchers to access and analyze anonymized data with state-of-the-art analytical tools and pipelines, facilitating a deeper understanding of brain development. The integration of high-performance computing infrastructure further enhances the ability to process and interpret complex datasets, which is crucial for studying the intricate processes of brain maturation.

Psychological research contributes to our understanding of how early adversities and individual resources impact brain functional connectivity. Studies have shown that early risk exposures, such as maltreatment, can significantly affect brain development. By examining these factors, researchers can identify critical periods during which interventions may be most effective [42]. Additionally, the study of approach-avoidant behaviors, which may be state-driven rather than trait-dependent, highlights the dynamic nature of emotional development and the importance of considering both environmental and biological influences [65].

Genetics and molecular biology provide insights into the underlying mechanisms of brain development. The recent development of large-scale sequencing technologies and whole-brain transcriptional atlases has enabled the multi-scale study of genetic, cellular, and molecular associates of human brain organization. However, translating genetic discoveries from animal models to humans remains challenging, emphasizing the need for interdisciplinary collaboration to overcome these hurdles [2]. Identifying prenatal and early-life resilience factors is particularly important given the neuroplasticity during the first decade of life. This knowledge can guide the implementation of evidence-based policies and interventions that have long-lasting and cumulative benefits [3].

Social sciences play a crucial role in understanding the broader context of emotional development. For example, maternal mental health and socioeconomic status are significant factors that influence a child’s emotional health. Approaches such as social, educational, and fiscal support can help reduce emotional and psychological stresses on women, particularly those of lower socioeconomic status. Comprehensive support systems, including fiscal assistance, mentorship, community support, and extended leave, are essential for promoting maternal and child well-being [31].

Furthermore, interdisciplinary research can address the limitations of current studies. For instance, the exploratory nature of some statistical analyses and the lack of correction for multiple comparisons highlight the need for robust methodologies and comprehensive data analysis techniques. By integrating knowledge from different disciplines, researchers can develop more accurate models and improve the reliability of their findings [66].

In summary, interdisciplinary approaches are vital for advancing our understanding of the maturation of brain regions associated with emotion. By combining insights from neuroscience, psychology, genetics, and social sciences, researchers can develop a holistic view of how early experiences and environmental factors shape emotional development. This comprehensive approach is essential for identifying effective interventions and promoting emotional health during the critical period from fetal development through the age of two.

## Data Availability

No data were collected or employed in this study.

## Abbreviations

The following abbreviations are used in this manuscript:

ANS	autonomic nervous system
CRH	corticotropin-releasing hormone
dACC	dorsal-anterior cingulate cortex
DMN	default mode network
fMRI	functional magnetic resonance imaging
HPA	hypothalamic–pituitary–adrenal axis
OFC	orbitofrontal cortex
PFC	prefrontal cortex
rs-fMRI	resting-state functional magnetic resonance imaging
SES	socioeconomic status
vmPFC	ventromedial prefrontal cortex
